# Hemoperitoneum Due to Dissection and Rupture of the Superior Mesenteric Artery in a Patient With COL3A1 Mutation

**DOI:** 10.1177/19253621241283723

**Published:** 2024-10-01

**Authors:** Fabio Tironi, Wijesinghe Lakmali, Jayantha Herath

**Keywords:** Forensic pathology, vascular Ehlers-Danlos disease, COL3A1 mutation, hemoperitoneum, superior mesenteric artery rupture

## Abstract

**Introduction:** Vascular Ehlers-Danlos syndrome (vEDS) is an autosomal dominant disorder that results from mutations in the collagen type III gene. It is a risk factor for medium-sized artery aneurysms, dissections, and ruptures. We report a case of hemoperitoneum due to medial dissection and rupture of the superior mesenteric artery related to vEDS. **Methods:** A full body CT scan and full three cavity autopsy was performed in a 47-year-old man with a history of an intermittent abdominal cramping for one week rand complex past medical history that included a sigmoid bowel perforation at age 20, and previous popliteal artery pseudoaneurysm rupture. Histology and genetic testing were performed. **Results:** The postmortem computed tomography and autopsy showed a significant hemoperitoneum due to a ruptured dissection of the superior mesenteric artery and branches, and multiple splanchnic artery dissections with renal and small bowel infarctions. Genetic testing revealed a heterozygous COL3A1 gene variant associated with Ehlers-Danlos syndrome. Death was attributed to hemoperitoneum due to medial dissection and rupture of the superior mesenteric artery due to arteriopathy. **Discussion:** The relatively young age and medical history correlate with the autopsy findings and genetic testing towards the conclusion of an arteriopathy consistent with vEDS.

## Introduction

Vascular Ehlers-Danlos syndrome (vEDS) is an autosomal dominant disorder that results from mutations in the COL3A1 gene, which encodes the pro-alpha1 chain of type III procollagen (1). Patients with pathogenic mutations have a greater risk of medium-sized artery aneurysms, dissections, and ruptures (2).

Visceral artery abnormalities are relatively rare clinical entities, although their detection is rising due to an increased use of cross-sectional imaging. Rupture is the most devastating complication and is associated with high morbidity and mortality (3).

We report a case of hemoperitoneum due to medial dissection and rupture of the superior mesenteric artery related to vEDS.

## Case Report

A 47-year-old man went to a hospital due to abdominal pain. He was complaining of a sudden onset of abdominal pain while defecating. He also described having a one-week history of intermittent abdominal cramping, attributed to constipation. His past medical history was of pyloric stenosis with pyloroplasty in infancy, scoliosis, bilateral inguinal hernia repair, a sigmoid bowel perforation of unknown etiology at age 20 requiring partial colectomy, a right popliteal artery pseudoaneurysm rupture with compartment syndrome requiring decompression fasciotomy and vascular repair. He also had bruised very easily and had some degree of joint hypermobility. His family history includes a father with a ruptured right femoral artery in his forties and previous surgery for an aortic aneurysm.

Computed tomography in the hospital showed hemoperitoneum with blood collections in the left upper quadrant and pelvis. A computed tomography angiogram revealed suspected large vessel vasculitis involving the celiac axis, bilateral renal arteries, and the splenic artery. He also had multiple renal infarctions. The bleeding was presumed to be due to a small branch aneurysm rupture not seen on imaging.

He was seen by a rheumatology service who suggested that he had a genetic connective tissue disease, favored Ehlers-Danlos Syndrome. He was initiated on steroids and discharged home. On the same day, he had been complaining of intermittent abdominal cramping throughout the day. In the early evening, shortly after going to the bathroom, he was found unresponsive by family members. Resuscitation efforts were unsuccessful.

**Figure 1.**
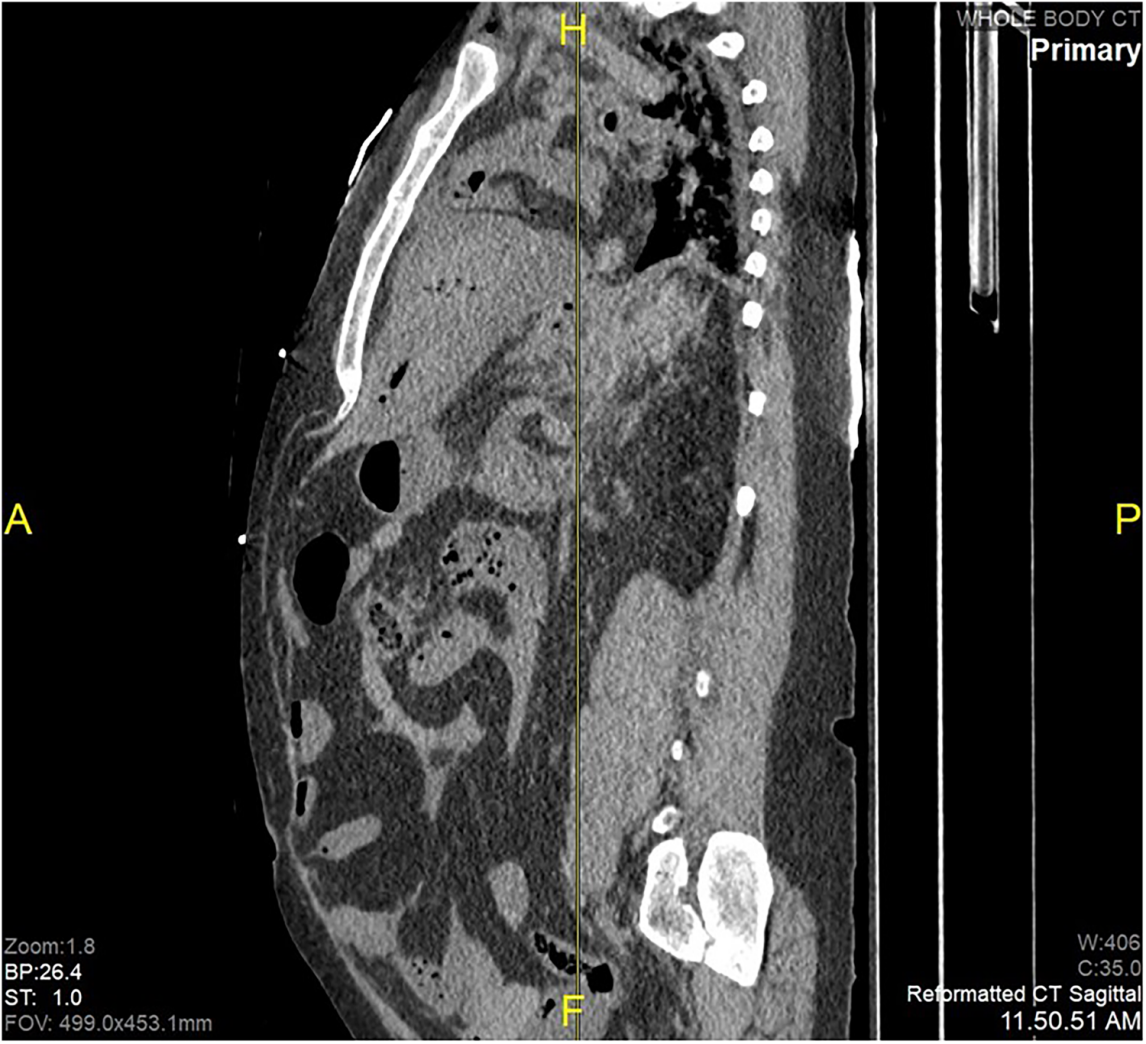
Sagittal reconstruction of PMCT revealing hemoperitoneum.

**Figure 2.**
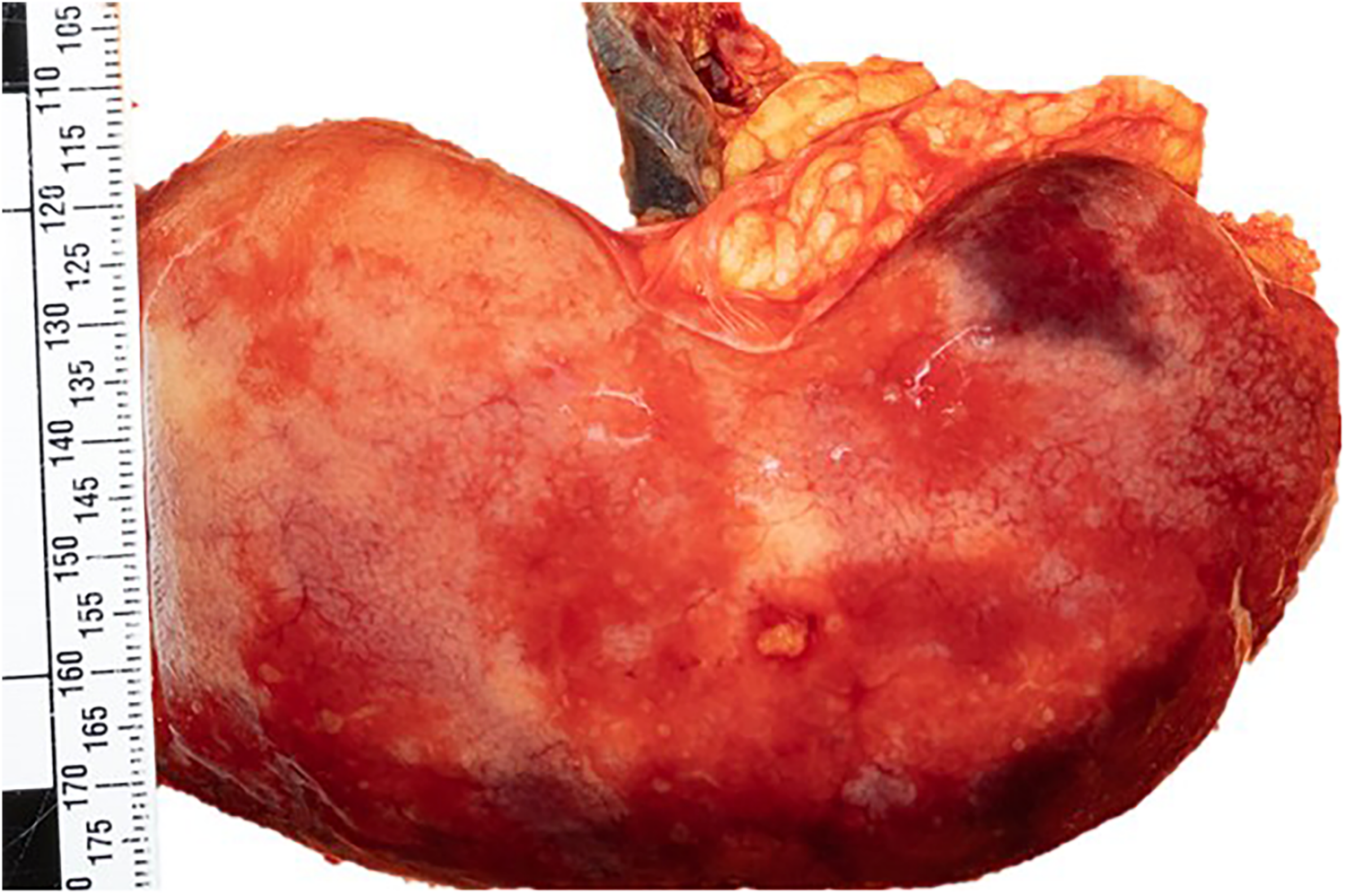
Patchy renal infarcts.

**Figure 3.**
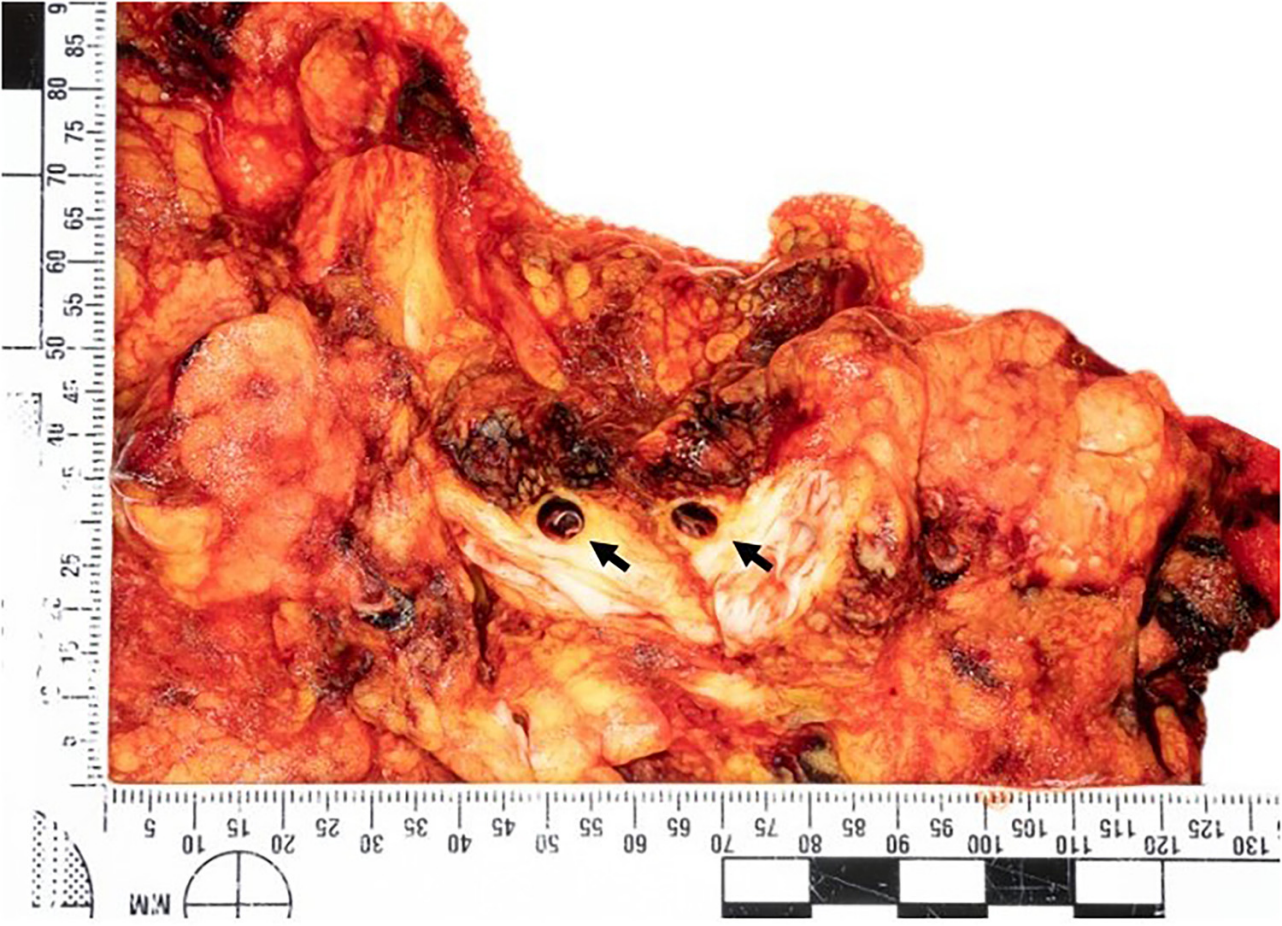
Section of the retroperitoneal fat showing dissection of the superior mesenteric artery (arrows).

Postmortem computed tomography revealed extensive retroperitoneal dissection and peritoneal hemorrhage (**Figure 1**). The autopsy examination was of an adult man with evidence of previous abdominal and right leg surgeries without trauma. The internal examination revealed a significant hemoperitoneum (more than 2L of partially clotted blood) due to a ruptured dissection of the superior mesenteric artery and branches. Further examination revealed an enlarged fatty liver and patchy renal infarcts due to bilateral renal artery dissections compressing the lumen (**Figure 2**), pancreaticoduodenal artery medial dissection, small bowel ischemic necrosis due to medial dissection of the main and the branches of the superior mesenteric artery. The main rupture site was identified as the superior mesenteric artery (**Figure 3**). The heart showed flame-shaped subendocardial hemorrhages of the outflow tract, indicating hemorrhagic shock due to bleeding (4).

PMCT, postmortem computed tomography.

**Figure 4.**
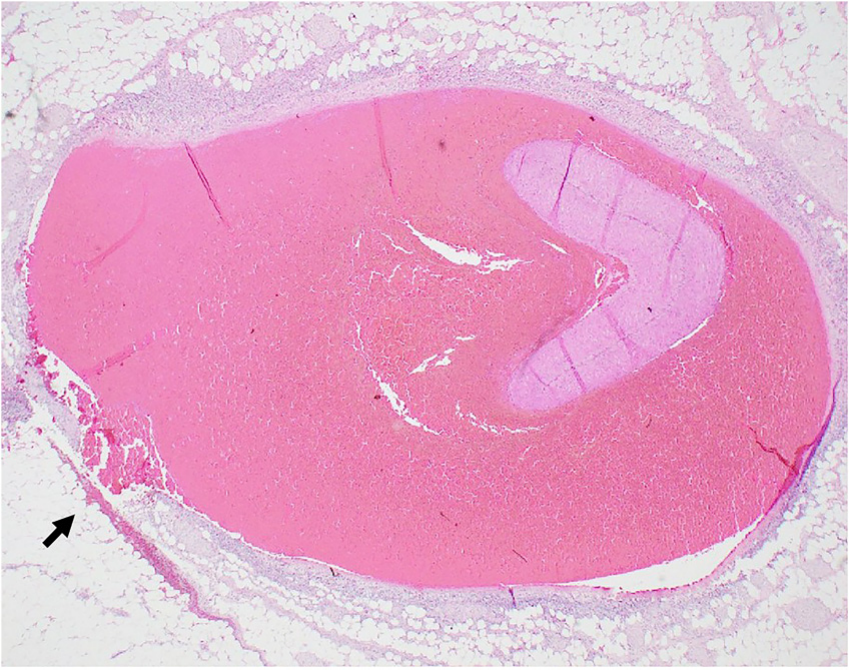
Branch of superior mesentery artery acute dissection and focal rupture (arrow). Hematoxylin-Eosin 12.5x.

**Figure 5.**
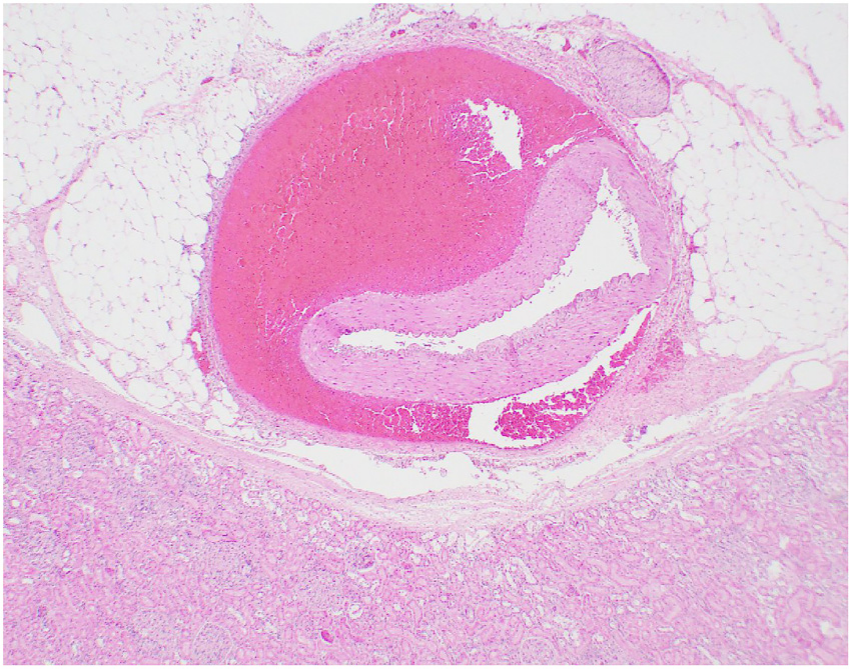
Renal Artery acute dissection. Hematoxylin-Eosin, 20x.

Microscopic examination confirmed medial dissection and associated hemorrhage of the superior mesenteric artery (**Figure 4**), dissection of the media and severe compression of the lumen of renal arteries (**Figure 5**) and the pancreaticoduodenal artery. The liver showed moderate fatty change.

Genetic testing revealed heterozygous COL3A1 gene variant c.1331G>A (p.Gly444Glu), a likely pathogenic variant associated with vEDS. Death was attributed to hemoperitoneum due to medial dissection and rupture of the superior mesenteric artery due to arteriopathy.

## Discussion

Peritoneal hemorrhages are not rare in forensic autopsies, but finding the source and the cause of the hemorrhage can be challenging in some cases. In the absence of a recognized trauma, other causes, such as vascular, gynecologic, iatrogenic and coagulation abnormalities, should be considered (5).

Hemorrhages due to the rupture of small intraperitoneal or extraperitoneal abdominal blood vessels in the absence of trauma or underlying pathology have been previously referred to as abdominal apoplexy (6). Nowadays, a diverse nomenclature has been used for cases with the overlapping characteristic of this cavitary hemorrhage related to a vessel rupture or a hidden cause, including idiopathic spontaneous intraperitoneal hemorrhage, idiopathic spontaneous rupture of a given artery (eg, splenic artery), spontaneous hemoperitoneum, spontaneous abdominal hemorrhage, hemorrhagic ascites, and idiopathic omental hemorrhage (6‐11). In many cases, the source and cause of hemorrhage remain unclear (12). Still, vascular diseases and less obvious sources of hemorrhages should be considered among the differential diagnoses, and a future review of this nomenclature could be recommended.

Several diseases can affect blood vessels, notably atherosclerosis, vasculitis, and connective tissue disorders (13). Vascular causes for hemoperitoneum comprise rupture of abdominal aortic aneurysm as the most common cause, followed by iliac and visceral artery aneurysms (7). In a series of living patients diagnosed with splanchnic artery aneurysms, the locations observed were splenic artery (55.4%), celiac trunk (27.0%), superior mesenteric artery (17.6%), hepatic (12.2%), pancreaticoduodenal and gastroduodenal arteries (4.1%), and gastric and gastroepiploic arteries (1.4%) (14). Among the medium-sized vessel vasculitides, polyarteritis nodosa is a known cause of intraabdominal bleeding from intraperitoneal rupture of hepatic, splenic, and/or renal (micro) aneurysms (15). An even rarer cause of abdominal vascular rupture is the portal vein aneurysm described in cirrhotic patients with portal hypertension. Localized at the level of main portal trunk, portal bifurcation and intrahepatic portal branches, the aneurysm may undergo spontaneous rupture of the portal trunk or one of its branches (16).

From the autopsy perspective, a thorough examination is needed since it can be challenging to locate the artery rupture site within a blood-infiltrated tissue (6). In this context, microscopic examination of splanchnic arteries can be elucidative. Diverse phenotypes of fibromuscular dysplasia and segmental mediolytic arteriopathy may result in an aneurysm, dissection, or rupture and could be suggestive of collagen mutations (17‐19).

Genetic abnormalities in the COL3A1 gene have been linked to vEDS (Type IV EDS) and detected in vascular rupture and hemoperitoneum cases (20). The genotype–phenotype associations of the gene variants confirm that vEDS is a severe early-onset disease resulting in arterial complications, predominantly local dissections, and aneurysms at the iliac, renal, and carotid arteries (80% with multiple locations); digestive complications as spontaneous colonic perforation in 85% of the cases; and obstetrical event in 12% of the cases in women (21). Subgroups of variations on the COL3A1 gene may present with a later-onset and a milder phenotype, with no or very rare digestive complications (21). Regarding the pattern of arterial involvement, a study of 68 individuals with vEDS concluded that mutations that lead to a minimal amount of production of normal type III collagen presented multivisceral arterial involvement. In contrast, haploinsufficiency mutations that lead to the production of half the normal type III collagen had a high prevalence of aortic disease, presented clinical events at an older age, and had milder arterial disease (22). These differences may affect diagnostic strategy, genetic counseling, and clinical follow-up of patients with vEDS (21).

Assuming the most severe consequence of vEDS is the rupture of small–medium-calibre vessels, this diagnosis should be suspected in young people with unexplained arterial rupture, especially those with a family history of similar events (1). Assessment of first-degree family members by a specialist with inherited vasculopathy is also suggested to help exclude a potentially inherited disorder in other members of this family.

## Conclusion

The relatively young age, bowel perforation, right popliteal artery pseudoaneurysm and family history of a father with aortic aneurysm and ruptured femoral artery aneurysm were relevant clinical findings in our case. They correlate with the autopsy observation of dissected or ruptured aortic branches and with the genetic testing toward the conclusion of an arteriopathy consistent with vEDS.
